# DNP-PhD: A transformative shift

**DOI:** 10.1097/01.NPR.0000000000000424

**Published:** 2026-03-24

**Authors:** Tracie Kirkland, Michelle Zappas, Courtney J. Pitts, Cheryl Thaxton, Kim Posey, Leonie DeClerk

**Affiliations:** **Tracie Kirkland** is Associate Dean for Transformational Practice & Partnerships, Kenneth E. Morehead Endowed Chair in Nursing, and Associate Professor at the University of Nebraska Medical Center College of Nursing in Omaha, NE.; **Michelle Zappas** is the director of the Master of Science in Nursing/Family NP program and an Associate Teaching Professor in the Department of Nursing, Suzanne Dworak-Peck School of Social Work at the University of Southern California in Los Angeles, CA.; **Courtney J. Pitts** is Associate Dean of Graduate Programs & Practice and Associate Professor at University of Kentucky in Lexington, KY; **Cheryl Thaxton** is Associate Dean/Chair for Graduate Studies and Professor at University of North Texas Health Science Center, Fort Worth College of Nursing in Fort Worth, TX.; **Kim Posey** is a Director of Graduate Nursing and Associate Professor of Professional Practice at Texas Christian University in Fort Worth, TX.; **Leonie DeClerk** is an Associate Professor at University of Arkansas for Medical Sciences College of Nursing in Little Rock, AR.

**Keywords:** DNP-PhD scholar, nurse scientist, synthesis, transformative, translation

## Abstract

Completing both Doctor of Nursing Practice (DNP) and Doctor of Philosophy (PhD) in Nursing degrees offers benefits and challenges. Integrating these programs enhances nurses' skills as clinicians and scientists, necessary for nurse practitioners in academic and clinical settings. An increasing number of nurses combine DNP and PhD degrees. This descriptive review evaluates this transformative shift in nursing education and practice. It offers perspectives to understand this change and discusses the significance of the shift. The review highlights role changes and opportunities related to education, scholarship, and practice. Additionally, it addresses policy implications, theoretical considerations, and organizational changes necessary to support this transformation.

The health care landscape is changing. Emerging technologies and advances in science have increased population longevity. In conjunction, the complexities of disease processes, along with increased chronic morbidity, require nurses and advanced practice registered nurses (APRNs) to be prepared to think critically. Social drivers that lead to poor health outcomes increase the need for nurses to be prepared at the highest level of training, with the intention of generating new research and translating and synthesizing it into practice. Nurses with both Doctor of Nursing Practice (DNP) and Doctor of Philosophy (PhD) in Nursing degrees are well positioned to accomplish this goal. The purpose of this paper is to provide a descriptive overview of transformative practices that uniquely blend the DNP- and PhD-prepared scholar.

## BACKGROUND

Nurses are encouraged to obtain terminal degrees: the DNP, Doctor of Nursing Science (DNS), and PhD in Nursing. The DNP is explicitly identified as the terminal degree for practice, encompassing the scholarship of integration and application. The PhD and DNS degrees involve the development of new knowledge and the refinement of existing knowledge. Recent data confirm decreased enrollment in PhD programs, from 5,045 in 2014 to 4,223 in 2024, and increased enrollment in DNP programs, from approximately 20,000 in 2014 to 42,767 in 2024.^1^-^3^ There is only one DNS program in the US, and no data on enrollment are available.^1^

In addition to the traditional routes for obtaining terminal degrees, a new option is emerging that combines the DNP and PhD. Some nurses who hold a DNP degree are choosing to enroll in PhD programs, whereas some PhD-prepared scholars are opting to pursue a DNP degree. Those without prior doctoral degrees may seek a program leading to a dual PhD/DNP degree. In 2024, eight programs awarded a dual PhD/DNP degree upon graduation, and several colleges now offer DNP-to-PhD bridge programs.^1^ These diverse pathways for completing terminal nursing degrees represent an innovative and transformative shift in nursing education.

## EDUCATIONAL PREPARATION

The transition from DNP preparation to PhD preparation requires a strong foundation in advanced clinical practice along with the development of research competencies. In 2010, the American Association of Colleges of Nursing (AACN) published a report entitled *The research-focused doctoral program in nursing: Pathways to excellence*.^4^ This report established the PhD as the `highest academic degree in nursing and outlined the factors that influenced its development, as well as the expected outcomes of PhD nursing programs.

In 2022, AACN released a revised report that addressed the evolution of PhD programs in relation to their foundational principles, as it relates to its underpinnings, recruitment and retention strategies, educational pathways, and overarching goals for students and faculty.^5^ The report stated that approximately 44% of students matriculating in PhD programs considered a DNP program prior to committing to a PhD program. The report acknowledged limitations and the need to collect data for analysis to glean more about the decision-making process. Although it does not provide specific guidelines for preparing DNP-prepared students for PhD studies, it notes that “students transitioning from the DNP program must identify a research focus for their dissertation as well as its proposed target population, anticipated research questions and methods, and professional goals for science and practice.”^5^

To date, there is limited literature addressing the educational preparation of DNP-prepared students enrolled in DNP-to-PhD programs. Of the 39 AACN member schools that offer opportunities to obtain dual nursing doctoral degrees (PhD and DNP), eight (20.5%) offer dual PhD/DNP degree programs, 35 (89.7%) offer DNP-to-PhD pathway programs, and five (12.8%) offer both dual PhD/DNP degree programs and DNP-to-PhD pathway programs.^6^ A random selection of curricular plans of seven DNP-to-PhD programs from across the country provided insight into the common characteristics of the programs. The average length of a DNP-to-PhD program of study was 3 years with an average of 36 credit hours required. The highest number of required credit hours found was 54. Each program offered a full-time plan of study, with most also offering a part-time plan of study. Programs were offered in online or hybrid learning format options that ranged from weekly online synchronous class sessions to 2-4 days of in-person intensives each semester. Each of the DNP-to-PhD programs reviewed granted course equivalency credits on a case-by-case basis. The number of course equivalency credits accepted for transfer by the programs ranged from 9 to 27 credit hours. Often, courses were historically identified as overlapping in the DNP or PhD curricula. Such courses included, but were not limited to, ethics, health policy, social determinants of health, leadership, measurement and testing, and interprofessional collaboration.^7^

At the granular level, curricula across programs commonly included foundational, research-focused, and dissertation courses. Programs of study often began with foundational, theoretical, and philosophical courses to introduce students to the scientific underpinnings of the degree. These courses were usually followed by introductory courses in qualitative and quantitative research methods, as well as in statistical and state-of-the-science courses. As the plan of study progressed, electives and research-focused courses that reflected the available expertise of faculty at each respective institution were threaded throughout the curriculum. Examples of these courses included, but were not limited to, measurement and testing, grant writing and publication, research practicum, middle range theory, phenomenology, and leadership courses. The requirement of electives or cognates offered opportunities to complete PhD-related certificates in areas such as health policy, or opportunities for interprofessional learning, for example, with faculty from public health and social work. Each program included a comprehensive qualifying exam (an important milestone associated with progression toward completion of a PhD program), a dissertation proposal, and a dissertation defense, aligned with the format of the traditional PhD program. At minimum, a student may have to complete 6 credit hours of a dissertation course, with the option to then participate in a continuation course. The highest number of credit hours for dissertation courses in the reviewed programs was 24.

Understanding the educational preparation of DNP-to-PhD students is crucial for ensuring that they are equipped to navigate the shift from practice-focused skills to rigorous scientific inquiry. Recognizing this preparation also helps ensure that individuals who are dually DNP- and PhD-prepared can effectively utilize their clinical expertise, advance nursing science, and succeed in academic roles. DNP-to-PhD programs have the potential to develop scholars who are equipped to transform practice, inform policy, and improve health outcomes through research.

## SIGNIFICANCE

The evolution of the DNP and PhD scholar represents a significant moment in the nursing profession. Established academic, practice, and policy structures are being reexamined to address the evolving demands of health care. Today's health systems are confronted with ongoing health inequities, rapid technological advancements, workforce shortages (both at the bedside and in academia), and increased expectations for evidence-based outcomes and efficiency.

As of 2024, among nursing schools offering baccalaureate and graduate degrees, the overall faculty vacancy rate was 7.9%, with the majority of open positions (more than 80%) requiring or preferring candidates with doctoral preparation.^8^ Although the registered nurse (RN) workforce is projected to grow from 3.1 million to 3.3 million between 2022 and 2032—a total increase of 177,440 nurses—the growth is not enough to meet workforce needs.^9^ According to the US Bureau of Labor Statistics (BLS), the RN workforce is expected to increase by only 6% during this period. Furthermore, when accounting for retirements and workforce departures, the BLS estimates that the US will need approximately 193,100 additional RNs each year through 2032 to maintain adequate staffing levels.^9^ In this context, the traditional distinction between knowledge generation (represented by the PhD) and knowledge application (represented by the DNP) has become inadequate in addressing the complex challenges faced by patients, communities, health care providers, and broader health systems. Nurses with both DNP and PhD degrees are uniquely equipped to bridge the gap between research and practice. Their combined expertise enhances the nursing workforce in several crucial ways. First, they contribute to a pipeline of nurse scientists who generate high-quality research grounded in clinical realities. Second, their skills in implementation and system-level thinking are critical for translating scientific evidence into sustainable practice innovations, particularly as health systems shift toward value-based care and population health models. Third, holding both degrees enhances their leadership abilities across academic, clinical, and policy domains, enabling them to (a) guide organizations through rapid change, (b) redesign educational programs, (c) influence health policy, and (d) foster interprofessional collaboration.

At the institutional level, the DNP-to-PhD pathway challenges outdated academic structures that limit the advancement of practice scholars and highlight disparities in workload and research support. Dually prepared faculty can expedite the dissemination of evidence to patients more quickly or increase the scientific rigor of innovations and interventions. Without this adaptation, there also may be worsening health inequities and system-level outcomes. The emergence of these uniquely prepared professionals signifies a broader transformation within the nursing discipline, as nursing increasingly recognizes that advancing health, reducing disparities, and redesigning care systems demand multiple complementary forms of scholarship.

Those who hold both degrees are not only academically versatile but also serve as vital connectors during periods of change in the field. Their combined DNP and PhD education enables them to integrate theory development with real-world implementation, identifying where research fails to inform practice and where clinical experience reveals unresolved scientific questions. By leading interdisciplinary research that spans effectiveness, equity, systems, and translational science, dually DNP- and PhD-prepared individuals exemplify the evolving view that practice and scholarship are mutually reinforcing rather than separate domains.

Within this context, the significance of the DNP-to-PhD shift extends beyond individual career trajectories. It represents a reorganization of nursing science toward more integrative, equitable, and practice-relevant forms of knowledge production. This shift provides a model for developing the disciplinary infrastructure required to address unique and complex public health and health care challenges.

## LIMITATIONS

Barriers exist related to this emerging phenomenon. There may be role confusion for internal and external stakeholders in academia, clinical, and within industries.^10^,^11^ Intentional efforts are required to clarify degree-specific roles, expectations, and terminology associated with DNP and PhD preparation.^10^ The tripartite faculty expectations of education, practice, and scholarship complicate the development of a clearly defined area of expertise and may create ambiguity in tenure and promotion pathways.^10^

University policies and financial fiduciary constraints further complicate dual and bridge pathways, particularly for students pursuing concurrent clinical and research preparation.^11^ A blueprint for mapping college credits and acceptance of credits requires collaborative planning. Programmatic time constraints also represent a substantive limitation. Dual preparation often requires completion of an extensive number of credit hours within compressed timelines, creating tension between advanced clinical hour requirements and sustained research training demands.^11^ Even in bridge or integrated models designed to reduce redundancy, students must simultaneously meet expectations related to clinical competence, dissertation development, and scholarly productivity, which may limit accessibility for working clinicians or those without strong institutional or financial support.^11^,^12^ Collectively, these limitations underscore the need for intentional program design, transparent role definitions, and institutional readiness, rather than assuming dual preparation alone resolves the practice-science divide.^10^,^11^

## A CONCEPTUAL FRAMEWORK FOR ORGANIZATIONAL CHANGE

To understand the DNP-to-PhD shift, we looked to Ernest Boyer's model of scholarship and Thomas Kuhn's theory of scientific revolutions. Boyer's model of scholarship underscores faculty's concern about the lack of recognition and reward for their work, which is characterized by four domains: discovery, integration, application, and education.^13^ This framework serves as a foundation for exploring the intersection of teaching, service, and research, which is evident in the combined skills of DNP-prepared PhD graduates. Kuhn's theory of scientific revolutions suggests that scientific advancement does not proceed in a linear, purely objective way but rather through cycles of stability and transformative upheaval.^14^ This theory popularized the concept of “paradigm shifts,” highlighting how science progresses through significant shifts in worldview rather than through gradual accumulation of knowledge. Kuhn also emphasized that no single paradigm could address all anomalies, which is particularly relevant in the evolving field of nursing.

While Boyer's and Kuhn's work help explain why the DNP-to-PhD shift is occurring, John Kotter's change theory explains how the field of nursing can respond to it. The model provides delineated sequential steps that illuminate urgency, build alignment, create vision, embolden power, and influence organizational cultural change.^15^ For faculty, urgency is related to promotion and tenure policies and process. A postulated concern can be found in the lack of a blueprint delineating the value and work that DNP-PhD-prepared faculty bring. The varied skill set creates potential challenges in determining appropriate evaluative methods during annual performance ratings. More importantly, the immediacy in integrating competency-based education and understanding the wins of a dually prepared faculty member is a pivotal shift in ensuring the maximizing of talent and efficiencies. A transformative shift is occurring in accommodating the skill set of DNP-PhD faculty that not only meets the tripartite mission of scholarship, teaching, and service but also maintains clinical practice.

A collective sense of urgency has emerged, driven by faculty shortages, health care complexities, demographic shifts, and technological advances. Dually DNP- and PhD-prepared nurses can help redesign doctoral pathways, reform promotion and tenure criteria, and build structures that support translational scholarship. By producing visible short-term solutions such as new bridge programs, implementation science initiatives, and interdisciplinary research teams, dually DNP- and PhD-prepared leaders can help infuse these changes into the culture of nursing education and the profession.

Finally, there is a pressing need to increase enrollment in PhD programs as decreasing enrollment is seen nationally compared to upward trends for DNP applicants. It is unclear whether this transformative shift of the increasing number of dually DNP- and PhD-prepared nurses will augment PhD enrollment and faculty workforce development. The fiscal effects on institutions of the combined degrees are also unknown. There are no current data to capture these outcomes; therefore, data on this topic will be valuable as the shift continues. Subsequent phases of this change will focus on alignment and achieving small victories. This is a continuous process and ongoing data collection will improve our understanding of the transition. Ultimately, how this change influences organizational culture and health outcomes must be evaluated to determine the value of the transition to DNP-PhD preparation. Although these factors remain to be fully realized, they open the door for significant structural changes within academic, clinical, and community systems.

## ROLE CHANGE OPPORTUNITIES

DNP graduates enter PhD bridge programs with advanced systems thinking, implementation expertise, and a strong practice background; these skills complement the theoretical depth and methodological rigor acquired through PhD training.^16^ The value of the combination of DNP practice experience and PhD scholarly research knowledge is demonstrated by the successful collaboration between DNPs and PhDs, wherein new knowledge is translated and synthesized to improve health care practices and policy.^17^-^19^ The development of DNP-to-PhD bridge programs represents a meaningful and timely strategy to strengthen the nursing workforce, particularly in academic leadership, scientific inquiry, clinical innovation, and health care design.

### Academic leader

The DNP-to-PhD degree uniquely positions graduates for academic leadership roles, including university-level deans, provosts, and presidents. Their combined expertise supports meaningful engagement in academic leadership activities, including long-range strategic planning, program effectiveness evaluation, and data-driven decision-making. Their foundation in statistical analysis and outcomes evaluation enables them to interpret metrics associated with value-based care models. Additionally, they can assess the implications of these metrics for nursing curricula and clinical training, guiding academic programs to align educational priorities with the current expectations of health care delivery.

### Nurse scientist

In addition to leadership skills, the DNP-to-PhD trajectory strengthens the pipeline of nurse scientists needed to address national research priorities. Individuals prepared first as DNP clinicians often enter doctoral study with a deep commitment to patient-centered inquiry, a clear understanding of existing practice gaps, and the ability to generate research questions based on real-world clinical challenges. Through PhD preparation, they develop the scientific foundation necessary to design rigorous studies, secure competitive funding, and contribute to the nursing knowledge base.^20^ This dual training prepares nurse scientists to conduct translational and implementation research, fostering sustainable improvements at the system level.

### Clinician

As DNP-prepared nurses transition into PhD programs, they enhance their ability to integrate clinical insights with scholarly rigor. This combination allows them to engage in research that is both innovative and deeply rooted in the realities of patient care. There is a need for more of these cross-trained leaders who both generate and apply new knowledge.^21^ The clinician background of DNP-prepared nurses provides a crucial foundation for their development as scholars, practitioners, and future leaders. Their extensive clinical experience offers a nuanced understanding of patient needs, workflow constraints, interprofessional dynamics, and system-level barriers to effective care. This firsthand exposure to the complexities of real-world practice enables DNP-prepared PhD candidates to identify clinically relevant research questions that target and address genuine gaps in care delivery rather than focusing on purely theoretical concerns.

### Design innovator

Dually DNP- and PhD-prepared nurses leverage their advanced training to drive innovation at the intersection of practice, scholarship, and systems design.^16^ This includes applying principles of human-centered design, implementation science, and strategic planning to reimagine care delivery, build organizational partnerships, develop scalable interventions, and enhance patient/family experiences. These clinicians should take advantage of opportunities to identify clinical and operational gaps where artificial intelligence (AI) can provide AI-enabled decision support and predictive analytics and automation to improve outcomes, reduce errors, and optimize resource allocation.^22^ As design innovators, dually DNP- and PhD-prepared nurses are increasingly positioned to harness AI tools, machine learning, and digital health technologies to advance patient care and improve system efficiency.

## POLICY AND THEORETICAL IMPLICATIONS

The transition from DNP to PhD not only reshapes individual roles but also exposes critical gaps in academic policy and theory. Tenure and promotion decisions continue to prioritize the scholarship of discovery while areas where DNP-prepared PhD faculty often excel—the scholarship of application, integration, and teaching—are frequently undervalued.^13^ This imbalance creates structural inequities and limits the advancement of dual-prepared faculty.^13^

Policy changes are urgently needed to address these barriers. Current promotion and tenure processes frequently require DNP-prepared PhD faculty to restart at the assistant professor level, even when they bring years of teaching, service, and leadership experience to new positions. This practice not only creates salary disparities but also disincentivizes DNP-prepared faculty from pursuing a PhD. Additionally, nontenure faculty roles rarely include protected research time, which prevents faculty from securing grants, producing high-impact scholarship, and fully participating in research teams. Without reforms to workload models and promotion structures, institutions risk underutilizing the contributions of DNP-prepared PhD faculty.

The theoretical implications are equally significant. Nursing must move beyond the binary categorizations of the DNP as “practice-focused” and the PhD as “research-focused.” Instead, the DNP-to-PhD pathway illustrates the possibility of a scholar-practitioner identity in which discovery, application, integration, and teaching are interwoven. This trajectory calls for a reconceptualization of what it means to be a nurse scholar. By following Kotter's eight steps of organizational transformation, academic institutions and professional organizations can more fully embrace diverse forms of scholarship, ensuring that the research-practice continuum is recognized and valued in promotion, tenure, and recognition systems.^15^

## CONCLUSION

The primary goal of nursing is to enhance health, generate evidence, reduce disparities, and transform health care systems. Achieving this goal requires various types of scholarship. PhD-prepared nurses focus on developing new knowledge, theories, and methodologies, whereas those with DNP degrees concentrate on implementing, evaluating, and applying this knowledge into practice. If either role is missing, there is a risk that important evidence may not reach patients, or that innovations in practice may lack the necessary scientific rigor.

By examining the experiences of the dually DNP- and PhD-prepared nurse through the lens of Kuhn's paradigm shift framework, we can better understand the ongoing transformation in nursing science.^14^ This analysis highlights the legitimacy of diverse scholarly contributions. Additionally, Kotter's change theory offers a structured approach for initiating change in the nursing field, emphasizing the urgency for this transformation.^15^
